# Successful Pregnancy Outcome After Laparoscopic Sacrohysteropexy for Pelvic Organ Prolapse

**DOI:** 10.7759/cureus.13087

**Published:** 2021-02-02

**Authors:** Subha R Samantray, Ipsita Mohapatra

**Affiliations:** 1 Obstetrics and Gynecology, Prathima Institute of Medical Sciences, Karimnagar, IND; 2 Obstetrics and Gynecology, All India Institute of Medical Sciences, Kalyani, IND

**Keywords:** pelvic organ prolapse, laparoscopic sacrohysteropexy, pregnancy, caesarean section, pop-q

## Abstract

The protrusion of pelvic organs and their associated vaginal segments into or through the vagina is called pelvic organ prolapse (POP). In recent times, a larger number of women of reproductive age group are presenting with complaints of POP, seeking treatment for POP along with the preservation of the uterus. These groups of patients may plan for pregnancy in the future. There is limited data on successful pregnancy, delivery and long-term outcome after sacrohysteropexy. We present here the management of a case of pelvic organ prolapse quantification (POP-Q) stage-III uterovaginal prolapse who underwent laparoscopic sacrohysteropexy and later on conceived and delivered by cesarean section. The uterus remained well-supported at follow up of one year after delivery.

## Introduction

The protrusion of pelvic organs and their associated vaginal segments into or through the vagina is called pelvic organ prolapse (POP). Although the peak incidence of the problem occurs in the post-menopausal age group, a larger number of reproductive age group women nowadays are presenting with complaints of POP, seeking treatment for POP along with the preservation of uterus. Although conservative treatments provide symptomatic relief, higher stage POP requires surgical methods of correction [1]. Young age at presentation and desire of future fertility has encouraged surgeons for uterine preserving prolapse surgeries [2]. Sacrohysteropexy is a method found to be useful and provide stronger apical support resulting in a lower recurrence rate [3]. Nowadays, minimal invasive laparoscopic route is preferred over conventional methods.

There is limited data on successful pregnancy, delivery, and long term outcome after sacrohysteropexy. We here present a case of a 27-year-old woman with pelvic organ prolapse quantification (POP-Q) stage-III of uterovaginal prolapse who underwent laparoscopic sacrohysteropexy with no complications postoperatively. Later she conceived after one year of the procedure and delivered at 38 weeks by cesarean section. The uterus remained well-supported at follow-up of one-year after delivery.

## Case presentation

**Figure 1:**
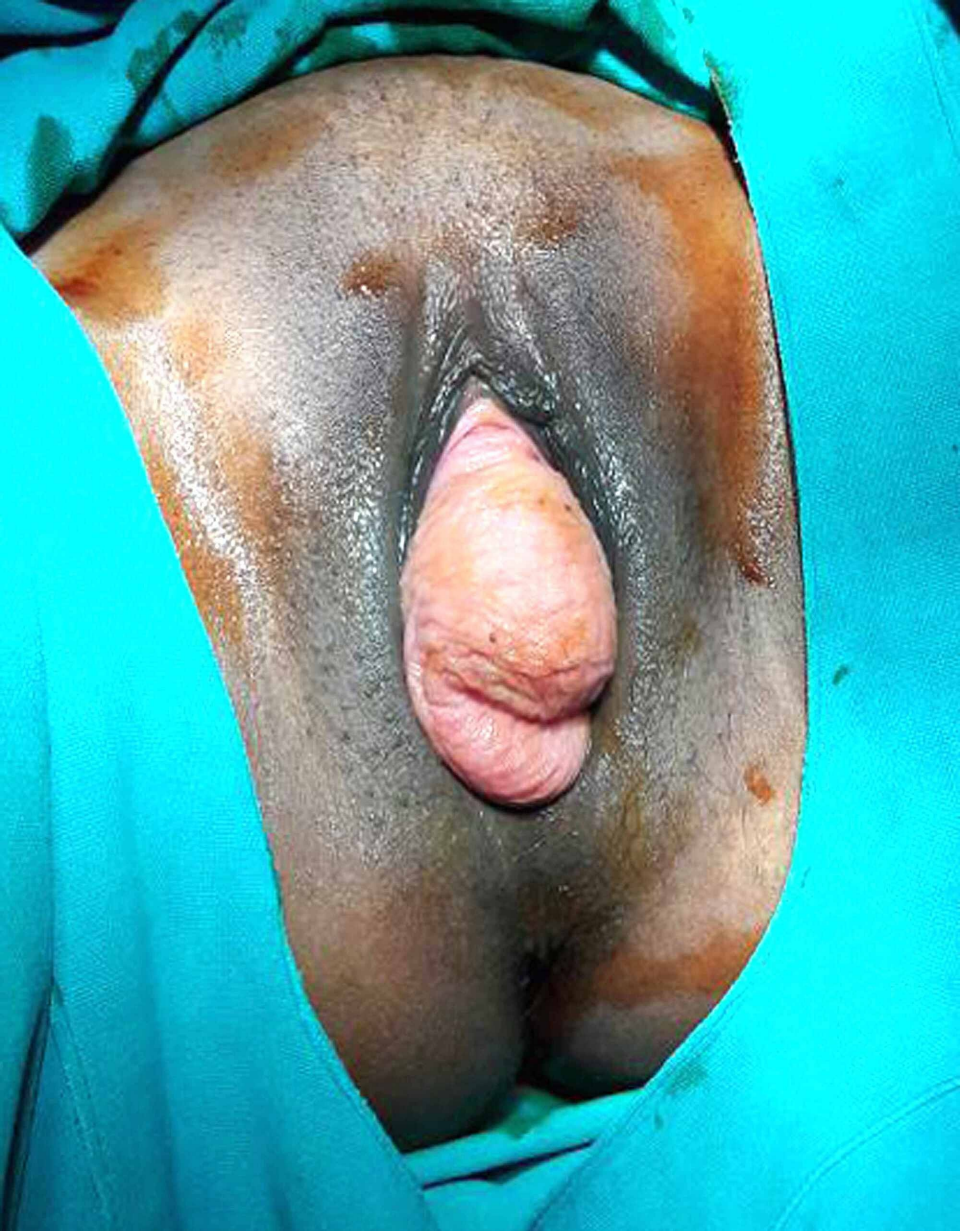
Stage III pelvic organ prolapse (preoperative)

A 27-year-old lady presented with complaints of mass per vaginum. She had an uneventful normal vaginal delivery four years back without any history of instrumental delivery or prolonged or precipitate labor. The initial POP-Q measurements were Aa: +3, Ba: +3, C: +7, D: +6, Ap: +6, Bp: +4, TVL: 7cm, PB: 3cm, GH: 4cm (stage III uterovaginal prolapse, Figure 1).

After proper counseling and informed consent, she was planned for laparoscopic sacrohysteropexy. The routine preoperative assessment revealed high fasting blood sugar (FBS) and postprandial blood sugar (PPBS) levels, and she was diagnosed with type 2 diabetes mellitus, for which she was started on tablet metformin (500mg) orally twice a day and injection insulin glargine (long-acting insulin) 10 units subcutaneously at night and subsequently. In the next week, metformin was increased to 1gm orally twice daily and insulin glargine 15 units subcutaneously as sugar levels were not controlled. Pap smear and endometrial biopsy revealed inflammatory Pap smear and proliferative phase respectively. Ultrasound abdomen and pelvis reported low lying uterus measuring 84x43mm, normal in size, contour, and echo-texture, no focal mass lesions, endometrial thickness 12mm, bilateral ovaries were normal. Daily vaginal packing with glycerine and betadine was done to prevent drying and ulceration.

**Figure 2:**
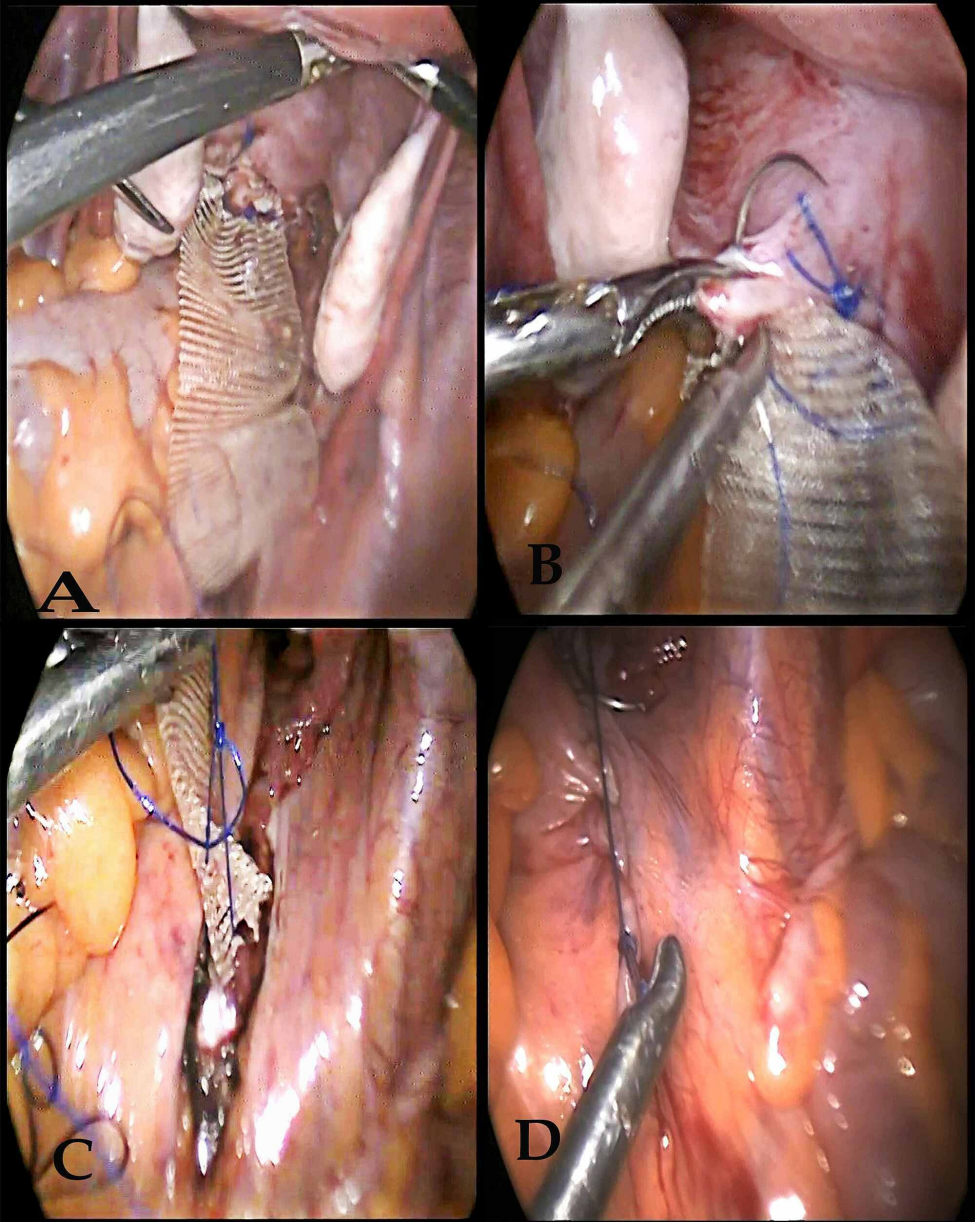
Intraoperative images (A) Fixation of mesh to the posterior part of the cervix. (B) Inclusion of the uterosacral ligament. (C) Fixation of mesh to anterior longitudinal ligament over the sacral promontory. (D) Peritonization of the mesh.

After the achievement of glycemic control, laparoscopic sacrohysteropexy was planned. A dose of injectable ceftriaxone (1gm intravenous) was given an hour before surgery. The patient was kept in dorsal lithotomy position under general anesthesia, a four-port laparoscopic entry was made with a primary 10-millimeter supraumbilical port and two ancillary secondary 5-millimeter port on the left side and another 5-millimeter port on the right side of abdomen lateral to inferior epigastric vessels. Uterus, bilateral tubes and ovaries were found to be normal. The uterus was elevated with the aid of a uterine manipulator inserted vaginally. Sacral promontory was identified. Peritoneal dissection was done from the sacral promontory to expose the anterior longitudinal ligament of the first two sacral vertebrae with the utmost care taken to avoid any vascular injury. The peritoneal incision was extended caudally and medial to the course of the ureter till the posterior part of the cervix, through Pouch of Douglas. A 4-centimeter wide macroporous monofilament knitted polypropylene mesh (type 1) was sutured to the uterosacral ligaments bilaterally, and posterior aspect of the cervix using five interrupted polypropylene suture (No 1-0) and the other end was fixed to the sacral promontory by direct suturing of the mesh to the anterior longitudinal ligament in a tension-free manner and the excess mesh was trimmed. The mesh was retroperitonized and hemostasis was secured (Figure 2).

**Figure 3:**
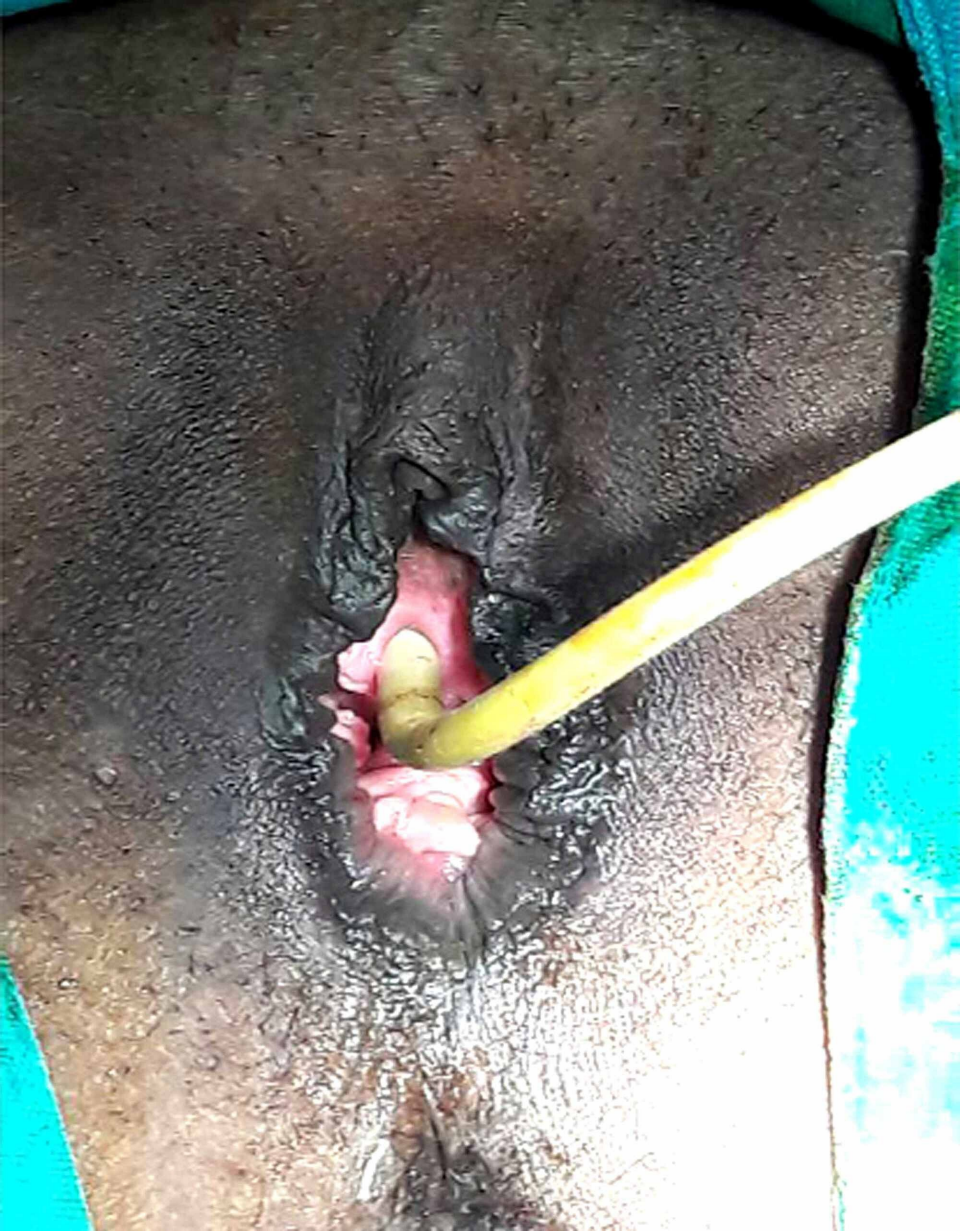
Postoperative image

An intraperitoneal drain was kept for 24 hours. Mesh being a foreign substance tends to induce infection and thus all possible steps were taken to prevent infection. Vaginal and rectal examination findings were found to be satisfactory at the end of the procedure (Figure 3).

On the third postoperative day, fasting and postprandial blood sugar levels were observed to be within the normal range. She was discharged on the sixth postoperative day and scheduled for follow-up at 15 days, third month, sixth month and then yearly thereafter. In the third month of follow-up, POP-Q measurements were Aa: −2, Ba: −1, C−4, D: −5, Ap: −3, Bp −2, GH: 4cm, PB: 3cm, and TVL: 9cm. After a year of surgery, she conceived spontaneously. All her antenatal visits were uneventful. Uterine artery Doppler study was normal at 32 weeks of gestation. At every visit, her FBS and PPBS were checked. She maintained glycemic control with tablet Metformin 500mg twice daily throughout her pregnancy. She was admitted at 37 completed weeks with complaints of dragging type lower abdominal pain. Elective cesarean section was planned at 38 completed weeks and she delivered a female child with a birth weight of 3.4kg. There were no complications intraoperatively. The patient was reviewed after 15 days and a vaginal examination revealed a well-supported uterus. The patient has been on monthly follow-up for one year now and has no complaints. One year after delivery, POP-Q measurements were Aa: −1, Ba: −2, C: −4, D: −5, Ap: −3, Bp: −2, GH: 4cm, PB: 3cm, and TVL: 8cm, showing no recurrence till the period of follow-up. 

## Discussion

Pelvic organ prolapse is a disorder of attenuated support and frequently observed in older women, after the fifth decade of life [4]. Due to more number of women opting for pregnancy at a later age, uterus preserving surgeries in patients with pelvic organ prolapse are becoming more common among women who desire future fertility and sexual activity [5]. Uterus preserving surgery has the advantage of maintenance of pelvic anatomy integrity, reduction in intraoperative blood loss, shortened operating time, and hospital stay so also contributes positively to the patient’s self-esteem, body image, confidence, and sexuality [6].

Laparoscopic sacrohysteropexy is a minimally invasive surgical procedure having the advantage of short operative time, minimal blood loss, short hospital stay, maintenance of good vaginal length and caliber to enhance sexual function and favorable functional outcome, beneficial for young women who want to preserve future fertility [3,7].

According to the document written on behalf of the urogynecologic society (AUGS) guidelines and statements committee, there are three case series [8-10] and two case reports [11,12] in the literature describing women who became pregnant after open or laparoscopic sacrohysteropexy [13]. Two more case reports have described women who underwent laparoscopic sacrohysteropexy and subsequently became pregnant [14,15]. Both of them were delivered by cesarean section. The follow-up study was uneventful in most of the above cases except a case reported by Lewis et al., recurrence of POP after two years of delivery occurred and later managed by robotic-assisted laparoscopic supracervical hysterectomy, sacrocolpopexy, and perineorrhaphy [12,15]. With limited data, it is difficult to predict whether the recurrence was due to pregnancy or other underlying factors [16]. Keeping these cases in mind, all women who plan for pregnancy after surgical repair of POP should be counseled properly about the chances of recurrent prolapse and safety concerns during pregnancy.

The mesh used in the surgery can theoretically hamper the uterine changes required to support the growth of the fetus due to contraction of the uterine arteries, specifically when it is wrapped around the isthmus [7]. To detect such changes, Doppler studies of the uterine arteries are advisable.

It has been postulated that posterior mesh may be the cause of pain during the third trimester because of the tension created over the mesh [13,15]. If the pain is very severe, the patient may need to be delivered. The delivery should preferably be done by cesarean section to prevent further injury to the pelvic floor [17].

Cesarean section was the preferred method of delivery among all available literature as it is logical to avoid disruption of reconstructed support [16]. Because of very limited data, it is not possible to make recommendations regarding the mode of delivery after surgery for pelvic organ prolapse [13].

Considering all these studies and results, the surgical treatment of young patients should be adjusted according to their symptoms and wish for future fertility. The key factors which are important for the prevention of recurrence are proper care during and after pregnancy, the patient’s lifestyle, and most importantly physiotherapy after delivery for the protection of pelvic floor muscles. There is also a consensus that conservative treatment has a minimal role in the treatment of POP in young patients [18].

The FDA has issued a warning about mesh in 2011 because of the seriousness of the problem with alloplastic materials like pain, discomfort, and sometimes erosions of the vaginal wall and intestinal obstruction secondary to the formation of adhesion [13,19]. But in hysteropexy, a narrow tape is used, which is reperitonized. So the chances of mesh erosion and other complications due to mesh are minimal.

Our patient was a young female who had not completed her family. So her prolapse was corrected by laparoscopic sacrohysteropexy. She was properly counseled regarding the chances of recurrent prolapse if she plans for pregnancy. Once she became pregnant, her complete pregnancy was monitored to detect any fetal growth restriction at the earliest. She was delivered by elective cesarean section at 38 completed weeks, and her follow-up till one year postpartum did not reveal any recurrence of prolapse or any other complication.

## Conclusions

Laparoscopic sacrohysteropexy is a standard procedure for the repair of POP in young patients desiring future fertility. It is a safe and effective procedure. Further, sufficiently powered randomized controlled trials with long term follow up are required to provide evidence-based decisions on the best way of treatment for POP in young patients.
